# Hyaluronic Acid Nanoparticles as Nanomedicine for Treatment of Inflammatory Diseases

**DOI:** 10.3390/pharmaceutics12100931

**Published:** 2020-09-29

**Authors:** N.Vijayakameswara Rao, Jun Gi Rho, Wooram Um, Pramod Kumar EK, Van Quy Nguyen, Byeong Hoon Oh, Wook Kim, Jae Hyung Park

**Affiliations:** 1 School of Chemical Engineering, Sungkyunkwan University, Suwon 16419, Korea; vijayrao@mail.ntust.edu.tw (N.V.R.); tings0609@nate.com (W.U.); pkumnair@gmail.com (P.K.E.); vquynguyents@gmail.com (V.Q.N.); obh6802@hanmail.net (B.H.O.); 2 Department of Chemical Engineering, National Taiwan University of Science and Technology, Taipei 10617, Taiwan; 3 Department of Molecular Science & Technology, Ajou University, Suwon 16499, Korea; rho8912@naver.com; 4 Department Biomedical Institute for Convergence at SKKU (BICS), Sungkyunkwan University, Suwon 16419, Korea

**Keywords:** hyaluronic acid, drug conjugates, polymeric nanoparticles, tumor-targeting, targeted-therapy, inflammatory diseases, type 2 diabetes, atherosclerosis

## Abstract

Owing to their unique biological functions, hyaluronic acid (HA) and its derivatives have been explored extensively for biomedical applications such as tissue engineering, drug delivery, and molecular imaging. In particular, self-assembled HA nanoparticles (HA-NPs) have been used widely as target-specific and long-acting nanocarriers for the delivery of a wide range of therapeutic or diagnostic agents. Recently, it has been demonstrated that empty HA-NPs without bearing any therapeutic agent can be used therapeutically for the treatment of inflammatory diseases via modulating inflammatory responses. In this review, we aim to provide an overview of the significant achievements in this field and highlight the potential of HA-NPs for the treatment of inflammatory diseases.

## 1. Introduction

Polymeric nanoparticles (PNPs) have received significant attention for healthcare applications since their enhanced permeation and retention (EPR) effects in cancer were observed by Maeda and co-workers [1]. Preferential accumulation of PNPs by the EPR effect in fast-growing, inflamed tissues like tumors has allowed the exploitation of PNPs for targeted delivery of imaging and therapeutic agents to diseased tissues. Several PNPs, including poly(ethylene glycol) (PEG)-conjugated liposomes, polymeric self-assemblies, and protein-based formulations are currently available on the market [2]. To reduce phagocytic uptake and renal clearance of PNPs, their surfaces have been decorated with hydrophilic polymers such as PEG, poly(vinyl alcohol), or polysaccharides [3]. The half-lives of PNPs have been increased by reducing their removal rates, providing additional opportunities to pass through fenestrated vasculatures, and accumulate in diseased tissues through the EPR effect. In addition to this passive targeting mechanism, the accumulation of PNPs can be further improved by active targeting through surface modification with ligands that specifically bind to the receptors on the target cells. It should be emphasized that, for high therapeutic efficacy, the therapeutic payloads need to be released from PNPs in a controlled manner after their preferential accumulation in the diseased tissues [4].

Use of microenvironment-responsive PNPs has emerged as a promising strategy for diagnostic and therapeutic delivery over the last decade [5,6]. These PNPs can be triggered by endogenous stimuli in the diseased tissues, such as pH [7], enzymes [8], cellular traction forces [9], reactive oxygen species (ROS) [10,11], or glutathione [12]. Ideally, they may exhibit prolonged circulation in the bloodstream and accumulate at the target site to show diagnostic or therapeutic effects. Although recent advances in materials science and engineering aimed to develop multifunctional PNPs, they are often prepared by multiple synthetic steps with poor yields and production cost too high for commercialization. Moreover, the complicated nanoparticular design makes them less biocompatible, reducing the chance for approval by the Food and Drug Administration.

**Figure 1:**
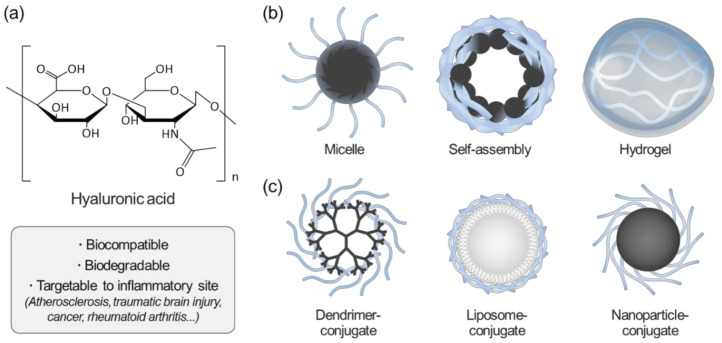
Overview of hyaluronic acid (HA)-based nanoparticles for biomedical applications. (**a**) Advantages of HA in biomedical applications; (**b**) Schematics of representative HA-based nanomaterials; (**c**) surface-modified nanomaterials using HA.

One of the representative biomaterials that can provide a simplified way of designing microenvironment-responsive PNPs is hyaluronic acid (HA), an anionic polysaccharide consisting of d-glucuronic acid and *N*-acetyl-d-glucosamine [13]. Owing to its biocompatibility and biodegradability, HA and its derivatives have been extensively investigated for biomedical applications (Figure 1). HA is also known as a ligand for cell surface receptors, including CD44, RHAMM, and lymphatic vessel endothelial hyaluronan receptor 1 (LYVE-1) [13]. In the human body, HA is catabolized by hyaluronidases (Hyals); HA-degrading enzymes such as Hyal-1, Hyal-2, and PH-20 [14,15]; as well as by oxidative stresses (OSs), including reactive oxygen species (ROS) and reactive nitrogen species (RNS) [16,17]. Notably, the levels of CD44, Hyals, and OSs are highly related to the progression of many types of inflammatory diseases such as atherosclerosis, cancer [18], and traumatic brain injury [19]. Because of these multifunctional benefits, HA has rapidly grown into one of the most popular biomaterials for targeted drug delivery and tissue engineering over the past two decades. A lot of studies have highlighted interesting functions of HA for its biomedical applications in various areas, including vascular disease [20], cancer [13,21,22,23,24,25], cancer metastasis [26,27], chondrocyte metabolism [28,29], lung injury [30,31,32], wound recovery [33,34], and diabetes [35,36]. In this review, our general focus is on healthcare applications of HA-NPs for various inflammatory diseases.

## 2. Characteristics of HA

### 2.1. Physicochemical Properties of HA

HA, the main component of the body’s extracellular matrix, is abundant in skin, synovial fluid, and vitreous humor [37]. It can be obtained by extraction from animal tissues, microbial production, or enzymatic synthesis. In general, HA is commercially isolated from animal sources inside the synovial fluid, umbilical cord, skin, and rooster comb [38,39]. The pKa of the carboxyl group of HA is in the range of 3–4; thus, these functional groups are deprotonated in physiological conditions [40]. Therefore, HA is highly hydrophilic and can absorb a large amount of water via hydrogen bonding to form a viscous and elastic gel [41]. In an aqueous solution, the HA molecule is stabilized by hydrogen bonds parallel with the chain axis and consequently takes up a stiffened helical configuration, which gives the molecule an expanded coil structure [42].

**Figure 2:**
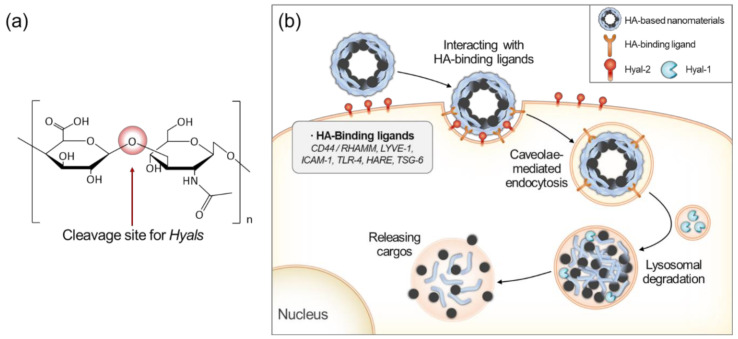
Endocytosis of HA-based nanomaterials. (**a**) Cleavage site of HA for Hyals; (**b**) Schematic of caveolae-mediated endocytosis of HA-based nanomaterials via HA-binding ligands.

It is produced by membrane-bound HA synthases, in which the molecular weight of HA ranges from 5 to 20,000 kDa in vivo [43,44]. Hyal-1 and Hyal-2 are key enzymes responsible for hydrolyzing the β-1,4-glycosidic bond of 3-*N*-acetyl glucosamine and 4-glucuronic acid (Figure 2a). This degradation of HA by Hyals is highly regulated in biological systems [45]. However, in inflammatory diseases such as cancer and rheumatoid arthritis, the degradation behavior of HA is dysregulated, resulting in the progression of the diseases [46]. In cancer, the degradation of HA by Hyals is highly affected by malignancy, angiogenesis, and metastasis [47,48]. In general, the high levels of Hyals are observed in various tumor types such as the brain, lung, bladder, and metastatic breast cancer [49].

CD44 is a cell-surface protein that plays a vital role in cell-cell interactions, cell adhesion, and migration [50]. It is often highly expressed on specific cells responsible for the pathogenesis of intractable diseases. For example, it is overexpressed on activated macrophages that play central roles in the development of atherosclerotic plaques, implying that CD44 is a useful target in atherosclerosis for molecular imaging and targeted drug delivery.

Interestingly, CD44 has a specific binding affinity for HA, suggesting that HA-based PNPs can be effectively taken up by cells that overexpress CD44 (Figure 2b). HA can also specifically bind to hyaladherins such as stabilin-2 (HARE) and TSG-6, which are abundant in atherosclerotic plaques and at sites of inflammation, respectively [51,52]. Therefore, HA-based PNPs are highly useful to target diseased tissue, based on specific interactions between HA and its receptors.

### 2.2. Pharmacokinetics of HA

Pharmacokinetics (absorption, distribution, and excretion) of HMW free HA was first determined in Wistar rats and Beagle dogs after single, oral administration of radioactively labeled HMW HA (1–1.5 MDa) [53]. Although 86.7–95.6% of radioactivity from radiolabeled HA was found to be eliminated by urinary and fecal excretion, incorporation of the radioactivity was detected in al tissues starting at 15 min and persisting for 48 h. HA was delivered to tissues via both lymphatic and blood circulation as well as a non-blood transport system [42,53,54]. Several studies have been also evaluated the plasma clearance, tissue distribution, and metabolism of intravenously administered HA using isotopes as tracers and reported that the injected HA was accumulated in the liver and degraded mainly by an efficient extrarenal system present in the liver [55,56]. Indeed, in the rat model, HMW HAs were mainly accumulated and remained throughout the 72 h experimental period in the liver, while LMW HAs were found in the urine [56], suggesting that both renal and hepatic systems play an essential role for the plasma clearance of HA. The plasma half-life of HA in normal human subjects after HMW HA injection was between 2.5 and 5.5 min, and its elimination was mainly extrarenal with the upper MW limit for renal excretion being 2.5 kDa [57].

### 2.3. Preparation of Self-Assembled HA-NPs

HA has been chemically modified to obtain its derivatives with proper physicochemical properties for specific applications in the biomedical field. Their properties can be substantially different from those of native polymer, but most have biocompatibility and biodegradability of indigenous HA. The chemical structure of HA includes sites for covalent modifications such as the carboxylic group, a hydroxyl group, and an -NHCOCH_3_ group. The carboxylic group allows chemical modification by amination and esterification [58,59]. HA can facilitate reductive amination via the aldehyde group at the reducing end of the polymer. Through deacetylation of the N-acetyl group, it is possible to recover an amine for an additional chemical reaction. Periodate oxidation generates dialdehyde groups via ring-opening of the d-glucoronic acid residue [60].

In an attempt to prepare self-assembled HA-NPs, the hydrophilic backbone of HA should be hydrophobically modified to obtain amphiphilic derivatives. For example, HA can be modified with fatty acids, bile acids, hydrophobic imaging agents, or therapeutics (e.g., paclitaxel (PTX) and chlorin e6 (Ce6)) [59,61,62,63]. Amphiphilic conjugates often self-assemble to form PNPs in an aqueous solution. PTX was conjugated to the HA backbone via an acid-cleavable ester linkage and formed self-assembled PNPs and allowed pH-dependent PTX release in an acidic tumor microenvironment, resulting in an enhanced cytotoxic effect to tumor cells [64]. HA was chemically conjugated with 5β-cholanic acid (CA) to prepare PNPs in aqueous conditions [65,66]. In another example, HA was labeled with fluorescent dyes or radioisotopes to investigate their potential as contrast agents for noninvasive PET imaging of plaque-associated macrophages in atherosclerosis [67]. Intravenous injections of HA exhibited short residence time in the body because of their rapid degradation in blood and efficient uptake by the liver endothelial cells [68]. To increase the biological half-life of HA, chemically crosslinked HA nanogels have been prepared since they are usually more stable than the physically crosslinked analogs. To prepare crosslinked HA nanogels, both of HA molecules and crosslinkers should be spatially localized within the nano-sized compartments [68]. The ionotropic gelation method was also studied to prepare HA-NPs based on the polyelectrolytic interaction between oppositely charged polymers. For example, chitosan (CS), a cationic polysaccharide, has been used to interact with negatively charged HA to prepare PNPs [69]. Vecchione et al. reported a coacervation process to prepare PNPs, composed of the chitosan core and an HA-shell, for multimodal imaging with MRI and optical imaging [70]. Various HA-NPs were prepared, based on electrostatic interactions with cationic polymers to encapsulate and to release the therapeutic payload for delivery of therapeutic drugs for active tumor targeting [71,72,73,74]. However, this type of PNPs were not structurally stable under physiological conditions. The ionic complexes of PNPs may be disassociated when the pH and ionic strength are changed. To prepare structurally stable PNPs, Han et al. reported biostable, mineralized HA-NPs by using calcium phosphate [75]. Interestingly, the resulting HA-NPs showed a rapid release of DOX in the mildly acidic solution, owing to dissolution of the mineral layer as the diffusion barrier. HA-modified mesoporous silica nanoparticles (MSNs) were also prepared for targeted drug delivery [76]. HA-modified MSNs were efficiently taken up by the cancer cells, compared to bare MSNs. Consequently, DOX-loaded HA-MSNs showed higher cytotoxicity to HCT-116 cells than free drug or drug loaded-MSNs.

## 3. Effects of HA-NP on the Inflammatory Response

In mammals, HA is found in heart valves, skin, skeletal tissues, vitreous of the eye, umbilical cord, and synovial fluid [77]. In its native form, such as that in normal synovial fluid, HA presents as a high-molecular-weight (HMW) polymer, with a molecular weight greater than 1000 kDa [30]. In tissue lesion and inflammation, HMW HA is altered into monocyte-adhesive matrices that energize immune cells at the injured site to produce inflammatory cytokines through interactions with cell surface receptors. This results in the secretion of enzymes and free radicals, which break the long-chain HMW HA molecules into lower-molecular weight (LMW) forms that have extraordinarily wide-ranging and often adverse biological functions due to the activation of differential signal transduction pathways [78]. Previous studies have shown that LMW HA fragments with proinflammatory, immunostimulatory, and proangiogenic features are essential as endogenous danger signals in initiating an inflammatory response [79]. In contrast, HMW HAs possesses protective anti-inflammatory effects by lowering gene expression and inducing proinflammatory cytokines. Initial studies showed that differential signaling pathways triggered by-products from the HA degradation process involve interactions between HAs and their HA receptors on cell surfaces, especially CD44. These opposing biological functions of HMW and LMW HAs were attributed to their ability to induce CD44 clustering on the cell surface by differential HA chain lengths. Recent studies have shown that CD44 has two conformational forms, and HA size-dependent conformational switching between the low and high-affinity binding states induces the clustering of CD44 [80]. A HMW HA has multivalent or repeated binding sites on CD44 receptors that induce CD44 clustering and, later, initiation of various downstream signaling pathways that are different from that of LMW HA, which is why it is considered an anti-inflammatory agent [81]. Self-assembled HA-NPs shielded by an HA outer shell, which presumably resembles HMW HA containing multiple CD44 binding sites that can induce CD44 clustering [82], have been shown to exhibit anti-inflammatory effects through the same mechanism as HMW HA [83].

### 3.1. Active Targeting of HA-NP to Macrophages

Macrophages play a crucial role in modulating a wide range of infectious and inflammatory diseases, such as tuberculosis, atherosclerosis, rheumatoid arthritis, type 2 diabetes (T2D), and multiple sclerosis [84]. They are involved in triggering and stabilization of inflammation, resulting in tissue damage. For instance, a large number of macrophages are sensitized and can infiltrate the cholesterol-rich sites of an injured aorta, inducing damage to the fibrous tissue by capping the plaques that are common in atherosclerosis [85]. An increase in the number of infiltrated macrophages in inflamed sites can be viewed as an indicator of the inflammation [86]. Hence, practical approaches for observing macrophages and designing therapeutic delivery systems to mediate macrophage activities became more important in diagnosis and therapy [86,87]. Recently, there has been more focused on the utilization of NP systems for biological applications (such as imaging and drug delivery) due to their nanometer dimensions and large surface area, which give them the potential to anchor diverse ligands [84]. The development of NPs with specific ligands that are explicitly recognized and taken up by macrophages is crucial to target macrophages actively.

HA receptors such as CD44, the major HA receptor in human bodies [88], are well known to be expressed in macrophages [50]. Recruitment of leukocytes and macrophages in atherosclerotic plaque development is likely related to the interactions between HA and CD44. Kamat and co-workers [89] have synthesized iron oxide magnetic NPs coated with HA (HA-DESPIONs) that could be efficiently taken up by activated macrophages due to native biological recognition between HA at the surface of NPs and HA receptor CD44, which is highly expressed on stimulated macrophages. In another study, HA-NPs prepared by reacting amine-functionalized oligomeric HA with cholanic ester showed dominant uptake by pro-inflammatory macrophages in vitro and exhibited a high binding affinity toward atherosclerotic plaque-associated macrophages in mice [67]. Strikingly, they found that the efficiency of macrophage-HA-NP interactions was strongly dependent on the disease stage, which was attributed to macrophage phenotypic switching. In other research conducted by Rho et al. [83], receptor-mediated cellular uptake of both LMW-free HA and LMW-free HA-induced pro-inflammatory genes were inhibited by HA-NPs in mouse primary bone marrow-derived macrophages (BMDMs) isolated from wild-type (WT) mice. However, these effects of HA-NPs were not observed in CD44-null (CD44^−/−^) BMDMs. Moreover, cellular uptake of HA-NPs was clearly detected in WT BMDMs, but not in CD44^−/−^ BMDMs, suggesting that the HA-NP-CD44 interaction is responsible for active targeting of HA-NPs to macrophages and HA-NPs can interrupt the LMW HA interactions with CD44 on macrophages.

In conclusion, self-assembled HA-NPs could act as an efficient platform to selectively target activated macrophages at disease sites due to their interactions with HA receptors, including CD44, overexpressed on these macrophages. This feature can be utilized to visualize disease sites through imaging techniques and/or to cure various inflammatory diseases, including T2D, atherosclerosis, and arthritis using empty HA-NPs themselves or as carriers for chemotherapy and, immunotherapy.

### 3.2. Effects of HA-NP in Macrophage Infiltration

**Figure 3:**
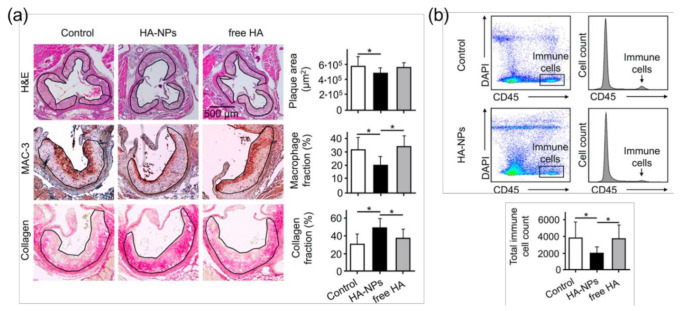
Hyaluronan nanoparticles improve plaque stability in atherosclerotic mice. (**a**) Representative images of aortic roots from mice that received either PBS (control, left image panel), HA-nanoparticles (NPs) (middle image panel), or free HA (right image panel) during a 12-week high-fat feeding period. The sections were stained with hematoxylin and eosin (H&E), macrophage-specific antibody (MAC-3), or Sirius red (collagen). Scale bar in the upper right image applies to all H&E-stained sections. Bar charts display mean plaque area (top), percentage of plaque area containing macrophages (middle), and collagen (bottom) in the aforementioned treatment groups; (**b**) Flow cytometric analysis of aortic arches of treated mice. The left panel shows representative cell scatter plots and histograms obtained for control (upper panel) and HA-NP-treated mouse (lower panel). The immune cells are defined as CD45-positive cells. The bar chart compares the total immune cell count in control, HA-NP-treated, and free HA-treated mice. In all bar charts, the symbol “*” indicates the significant difference at *p* < 0.05. Adapted with permission from [67]. Copyright 2017, American Chemical Society.

Under pathological conditions such as inflammation, HA attached to vascular endothelia arbitrates the mobility and extravasation of immune cells, and the HA-rich microenvironment at inflamed sites induces tissue penetration by immune cells [90]. In the research conducted by Beldman et al., infiltrated macrophages degraded the subendothelial matrix, whereas nanosized particles translocated to the atherosclerotic plaques through the transcellular migration pathway by exploiting intracellular vesicles [67]. In this work, the potential effect of long-circulating HA-NPs on the vascular glycocalyx, which primarily consists of HA, was anticipated [91]. Free HA is known to have a very short blood circulation half-life (3 to 5 min) [92]. However, HA-NPs might act as a circulating pool of HA owing to their favorable blood circulation kinetics and can be viewed as building constituents into the glycocalyx, limiting immune cell infiltration into injured sites [67]. The study also reported a reduction in the immune cell infiltration in aortic injury due to the atheroprotective effects of long-circulating HA-NPs in comparison with free HA (Figure 3). Another study carried out by Rho et al. has shown that the treatment of diet-induced obese (DIO) mice with HA-NPs suppressed macrophage infiltration into epididymal white adipose tissues (eWATs) and secretion of pro-inflammatory cytokines and chemokines [83]. In particular, cholanic acid-functionalized HA-NP (HA-CA NP) treatment reduced macrophage infiltration, as indicated by lower expression of the macrophage marker CD68 compared with the control. In addition, poly(ε-caprolactone)-conjugated HA-NPs (HA-PCL NPs) opposed macrophage infiltration into eWATs and relieved pro-inflammatory gene expression in DIO mice. The inhibitory effects of HA-NPs on macrophage infiltration are likely mediated by reduction of monocyte chemoattractant protein-1 (MCP-1, also known as CCL2), a key chemokine that recruits immune cells such as monocytes/macrophages into inflamed and injured sites by regulating their migration and infiltration [93] via blocking CD44. CD44 activation by LMW HA binding induces production of MCP-1 [94], and MCP-1 and CD44 levels were normalized by treatment with both HA-CA and HA-PCL NPs, leading to attenuated macrophage infiltration into eWATs [83]. In addition, the effect of CD44 blockade on macrophage infiltration was demonstrated by several researchers. Neutralization of CD44 by daily administration of anti-CD44 monoclonal antibody (mAb) in DIO mice led to a striking reduction of immune cell infiltration into the stroma of adipose tissues compared with control IgG-treated mice, and most infiltrating cells were positive for the macrophage marker MAC-2 and CD44, suggesting that most infiltrating cells in adipose tissues are CD44-expressing macrophages [95]. These effects of CD44 mAbs on macrophage infiltration into adipose tissues were supported by the research of Kodama [96], implying that CD44 mAbs neutralize CD44 receptors on macrophage surfaces, resulting in decreased macrophage activity. Consistently, genetic blockade of CD44 in DIO mice suppressed macrophage infiltration into adipose tissues.

### 3.3. Effects of HA-NP on Production of Pro-Inflammatory Cytokines and Chemokines

**Figure 4:**
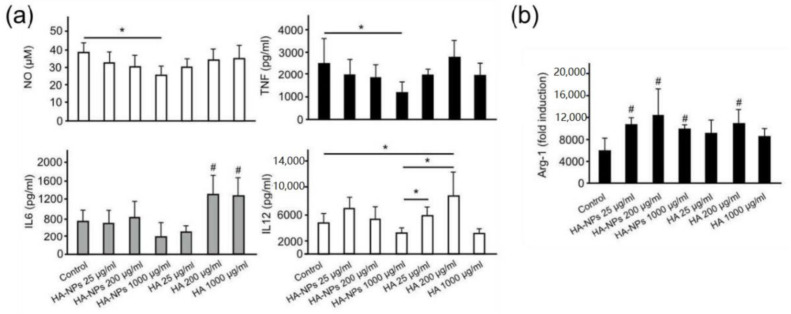
Anti-inflammatory effects of HA-NPs in bone-marrow-derived macrophages (BMDMs). (**a**) The concentrations of pro-inflammatory markers of nitric oxide (NO) (upper left), tumor necrosis factor (TNF) (upper right), interleukin-6 (IL-6) (lower left), and interleukin-12 (IL-12) (lower right), excreted by BMDMs that were stimulated with lipopolysaccharide (LPS) and interferon-γ (INF-γ) for 24 h (Control) or that were first pre-stimulated for 24 h with HA-NPs at three concentrations of 25 μg/mL, 200 μg/mL, or 1000 μg/mL and subsequently stimulated with LPS and INF-γ. Free oligomeric HA was used at the same concentrations and using the same protocol as mentioned above. In the lower-left panel, “#” indicates significantly higher concentration compared to control, HA-NPs (all concentrations), and HA at 25 μg/mL. In the lower right panel, significant differences “*” are shown only for control and HA-NPs 1000 μg/mL; (**b**) Expression of arginase-1 gene (Arg-1) (relative induction compared to naive macrophages), which is associated with the pro-fibrotic macrophage phenotype. BMDMs were stimulated either with interleukin-4 (IL-4) for 24 h (Control) or were first pre-stimulated with HA-NPs or HA for 24 h at the concentrations mentioned above and subsequently stimulated with IL-4. “#” indicates significantly higher values compared to control at *p* < 0.05. Adapted with permission from [67]. Copyright 2017, American Chemical Society.

**Figure 5:**
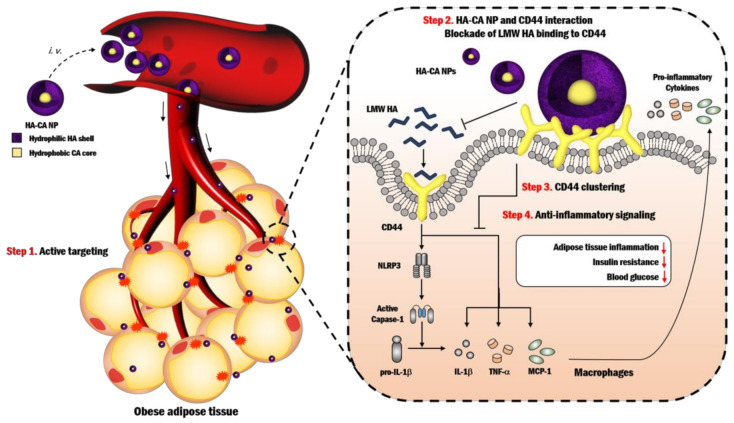
Schematic illustration of self-assembled HA-CA NPs for the treatment of type 2 diabetes (T2D). Adapted with permission from [83]. Copyright 2017, Elsevier.

As described, the biological activities of HA on inflammation are strongly influenced by its MW (chain length); HMW HA exerts anti-inflammatory effects [97], while LMW HAs promote pro-inflammatory response [98]. Apart from an increase of macrophage infiltration into inflamed and injured sites, LMW HA binding to CD44 induces expression and secretion of pro-inflammatory regulators including tumor necrosis factor-α (TNF-α) and interleukin-1β (IL-1β) [99], and these effects are attenuated by CD44 deletion and blockade [95,96]. Accordingly, Beldman and co-workers conducted an in vitro study in BMDMs and demonstrated a marked inhibition in the synthesis of the crucial pro-inflammatory regulators, nitric oxide (NO) and TNF-α, by a high concentration of HA-NPs (Figure 4) [67]. The same scenario was discovered for pro-inflammatory IL-6 and IL-12. In contrast to HA-NPs, HA oligomer showed no substantial effects on the production of NO and TNF, whereas it remarkably enhanced the production of IL-6 and IL-12. Strikingly, the increased expression of arginase-1 gene (Arg-1), an important marker of M2 anti-inflammatory macrophages, was observed as a consequence of HA-NP treatment. Recently, we reported anti-inflammatory functions for blank HA-NPs, HA-CA, and poly (ε-caprolactone)-conjugated HA (HA-PCL) NPs, without chemotherapeutic encapsulation through inhibition in the synthesis and secretion of pro-inflammatory cytokines and chemokines including TNF-α, IL-1β, and MCP-1 [83]. LMW-free HA increased the expression levels of TNF-α and IL-1β in WT but not CD44^−/−^ BMDMs, while this LMW-free HA-induced expression of the pro-inflammatory cytokines was attenuated by pre-treatment with HA-CA NPs. Accordingly, DIO mice treated with HA-NPs exhibited a reduction in the production of pro-inflammatory cytokines and chemokines in epididymal white adipose tissues (eWATs), leading to suppression of adipose tissue inflammation. The inhibitory effect of HA-NPs on the synthesis of pro-inflammatory cytokines and chemokines in macrophages is likely due to their ability to block CD44 activation via LMW-free HA binding to CD44 (Figure 5). The direct interaction between LMW-free HA and CD44 on infiltrated macrophages at the inflamed site played a key role in the inflammatory response by modulating pro-inflammatory cytokine production. This is further supported by the findings that expression levels of pro-inflammatory chemokines and cytokines, including MCP-1, MIP-1α, TNF-α, IL-1β, and IL-6, decreased in the adipose tissues of DIO mice treated with CD44 mAb [100].

### 3.4. Effects of HA-NP on NLRP3 Inflammasome Activation

The apoptosis-associated speck-like protein containing a C-terminal caspase recruitment domain (ASC; also known as PYCARD) is a key bipartile adaptor required for inflammatory processes. Incorporation of the ASC, nucleotide-binding domain and leucine-rich repeat protein 3 (NLRP3), and pro-caspase-1 forms the inflammasome complex. Activation of the NLRP3 inflammasome plays a critical role in the pathogenesis of many disorders, so-called inflammasomopathies consisting of autoinflammatory diseases [101,102], by inducing processing and secretion of IL-1β and IL-18 pro-inflammatory cytokines. Given that abundant pathogens or endogenous metabolites (ligands) have been reported to stimulate the NLRP3 inflammasome, the interaction between NLRP3 and bipartite adaptor ASC induced by these stimulants might be an attractive target for therapeutic approaches to inflammatory diseases. Recently, it has been reported that HA-NPs could be involved in the modulation of NLRP3 inflammasome activity under the pathological condition [83]. LMW-free HA-induced expression of NLRP3 was suppressed by pre-treatment with HA-NP. These were further confirmed in vivo. The eWAT in DIO mice had elevated gene expression of NLRP3, Pycard, Caspase-1, IL-1β, and IL-18 in conjunction with higher caspase-1 activity in the eWAT and IL-1β levels in both the eWAT and blood, whereas these parameters were reversed by HA-NP treatment.

## 4. Therapeutic Applications of HA-NP in Inflammatory Diseases

### 4.1. Obesity and T2D

More than 370 million people around the world suffer from T2D, which is a complex, metabolic, and polygenic disease. T2D is characterized by obesity-induced insulin resistance, and proinflammatory response in insulin-sensitive peripheral tissues, including liver, adipose tissue, and muscle, plays an essential role in the pathogenesis of T2D such as obesity-induced insulin resistance and associated complications [103]. To date, a number of crucial genes consistently implicated in the pathogenesis of T2D were successfully discovered through genetic association studies. In addition, the gene expression-based genome-wide association study (eGWAS), conducted across 130 separate experiments (a total of 1175 T2D case-control microarrays) to determine additional genes that are functionally associated with the molecular pathogenesis of T2D, identified CD44 as another leading candidate (P = 8.5 × 10^−20^) [96]. In agreement with this, genetic blockade of CD44 improved insulin resistance by suppressing adipose tissue inflammation in DIO mice, leading to normalized fasting plasma glucose level and macrophage infiltration into adipose tissue in DIO mice. Moreover, in humans, CD44 was overexpressed in inflammatory cells in obese adipose tissue, and its serum level was positively correlated with insulin resistance and glycemic control [104]. These results imply that CD44 is a key factor to cause insulin resistance via adipose tissue inflammation, and its blocking might be a potential therapeutic strategy for the treatment of T2D by breaking the links between obesity-induced insulin resistance and adipose tissue inflammation. Indeed, pharmacologic blockade of CD44 by anti-CD44 antibody treatment in DIO mice suppressed visceral adipose tissue inflammation compared to controls and decreased fasting plasma glucose level, weight gain, liver steatosis, and insulin resistance to levels equal to or better than those seen in therapies using the anti-diabetic drugs metformin and pioglitazone [95]. From another perspective, Rho et al. reported a novel therapeutic function of empty HA-NPs in adipose tissue inflammation, insulin resistance, and glycemic control [83]. Similar to anti-CD44 antibody treatment, [95,96] HA-NP treatment in DIO mice improved insulin sensitivity and normalized blood glucose levels by attenuating adipose tissue inflammation as indicated by reduced macrophage content, production of proinflammatory cytokines, and NLRP3 inflammasome activity in eWATs. Recently, it has been reported that HA-NP has anti-adipogenic and lipogenic effects in DIO mice, leading to a reduction in fat accumulation and body weight [105]. Treatment with HA-NPs in DIO mice reduced eWAT mass through suppression of adipogenesis and lipogenesis as indicated by decreased adipogenic and lipogenic regulators, while these effects were not observed in CD44^−/−^ mice, suggesting a potential anti-obesity strategy targeting CD44 by HA-NPs.

The effect of HA-NP in DIO mice has many similarities to cannabinoid 1 receptor (CB1R) antagonists in terms of hyperglycemia, insulin resistance, and adipose tissue inflammation. CB1R antagonists are potential therapeutic agents currently under early-stage clinical development for the treatment of obesity and its metabolic complications [106]. Similar to HA-NPs, CB1R antagonists improve obesity-induced insulin resistance by suppressing adipose tissue inflammation via the NLRP3 inflammasome as well as reduce fat accumulation and body weight in DIO mice [107,108]. Therefore, once safety, tolerability, and improved therapeutic efficacy of HA-NPs are proven, their clinical testing might be warranted for the treatment of obesity and its metabolic complications.

### 4.2. Atherosclerosis

Atherosclerosis is characterized by the accumulation of fatty deposits, cholesterol, calcium, and cellular debris (called plaques) in arterial walls. It is a leading cause of cardiovascular diseases such as myocardial infarction, heart failure, stroke, and claudication [109]. In addition, acute syndromes and mortality are involved in occlusive atherosclerotic plaques. Ruptured plaques have large lipid cores, thin fibrous caps, and numerous infiltrating macrophages. Commonly, atherosclerosis can be cured utilizing chemotherapeutic agents, which can relieve hypertension and hyperlipidemia or moderate hemostasis to reduce the risk of thrombosis-related complications. However, direct depression of inflammatory mechanisms underlying atherosclerotic progression was not found in these drugs. Even though high-density lipoprotein (HDL) mimetics and IL-1 receptor antagonists arose as a targeted therapeutic, their delivery to atherosclerotic lesions has been challenging [110]. Over the last several decades, tremendous efforts have been made to develop nanosystems for the diagnosis and treatment of atherosclerosis [111]. Targeted delivery to atherosclerotic lesions has remained a challenging issue over the previous two decades.

Researchers exploited HA-NPs to target atherosclerosis-associated inflammation [112]. Macrophages, which are important propagators of atherosclerosis and primary phagocytes in atherosclerotic plaques, express several HA receptors, such as CD44, ICAM-1, LYVE-1, RHAMM, and TLR-4 [113,114,115,116]. Macrophages are a highly dynamic cell population, and the tissue microenvironment strongly influences their molecular and functional profiles. Evaluation of these phenotypic alternations might have significant predictive and prognostic value in atherosclerosis. In a recent study, Beldman and co-workers reported brief research on interactions between HA-NPs and immune cells and how they are affected by atherosclerotic plaque progression [67]. Toward this goal, HA-NPs were prepared by reacting amine-functionalized oligomeric HA with cholanic esters labeled with a fluorescent or radioactive label. Radiolabeling of HA-NPs with 89Zr was performed for quantitative evaluation of plaque targeting efficacy. This research group studied the effect of assembled HA NPs on its biological function by inspecting atherosclerotic suppression efficacy induced by the NPs. Previously, our group reported that stabilin-2 (also called HARE), an HA receptor for endocytosis, is more overexpressed in atherosclerotic plaques than in normal vessels [117]. Interestingly, CD44 is also expressed on activated macrophages, which play key roles in the development of atherosclerotic plaques [118]. As HA is a ligand for both stabilin-2 and CD44, HA-NPs are expected to have the potential for active targeting of atherosclerotic plaques. Our group recently prepared self-assembled HA-NPs for atherosclerosis plaque targetability. The synthesized HA-NPs accumulated in the atherosclerotic region by an active targeting mechanism. The in vitro study showed selective uptake of HA-NPs by cells overexpressing HA receptors (such as stabilin-2 and CD44) [117].

### 4.3. Other Inflammatory Diseases

HA-NPs have been extensively investigated for other inflammatory diseases, including cancer and rheumatoid arthritis (RA). The functional groups (carboxyl, hydroxyl, and *N*-acetyl groups) of HA have been modified with hydrophobic molecules to prepare amphiphilic HA derivatives which can be self-assembled NPs to encapsulate therapeutic cargoes for inflammatory diseases. The drug-loaded HA-NPs can selectively and effectively accumulate into disease sites via the EPR effect or CD44 receptor-mediated endocytosis. Shin et al. reported the metal-phenolic network-coated HA-NPs to facilitate drug release in tumor microenvironments [119]. It was confirmed that the DOX release rate was high in the mildly acidic condition, mimicking the tumor microenvironment. In contrast, a much slower release was observed at pH 7.4. Owing to the dissolution of the metal-phenolic network on their surfaces, the DOX-loaded HA-NPs exhibited a higher cellular uptake at the mildly acidic conditions, suggesting their potential use as a drug carrier for targeted cancer therapy. In another study, Kim et al. reported HA-based NO-releasing PNPs to improve the anticancer activity of DOX. This PNPs may serve as a significant chemosensitizing agent in treatments of various cancers [120]. Recent advances on HA-drug conjugates and HA-based NPs for cancer therapy have been well described elsewhere [60].

Rheumatoid arthritis (RA) is an autoimmune disorder affecting 1% of the population worldwide. RA is characterized by chronic inflammation of the joint synovium and severe joint destruction. The exact pathophysiologic cause of RA remains uncertain. Although a wide range of drugs is available for RA therapy, the benefits are only temporary because of the adverse side effects, such as osteoporosis, hypertension, weight gain, and fluid retention. Hence, the development of an effective and safe carrier for targeted delivery would be desirable. As the HA receptor is over-expressed on synovial lymphocytes and macrophages of RA patients, it was expected that HA-NPs have the potential drug carrier for RA therapy. Shin et al. reported an HA-MTX conjugate, which has exhibited enhanced uptake efficiency in murine macrophages through interactions between HA and CD44 [121]. Due to the pH-sensitive nature of the conjugate, MTX was released at the inflamed synovial tissue in RA. In another report, Alam et al. prepared the HA-based pH-responsive mineralized PNPs for MTX delivery to target the inflamed joint [122]. The calcium phosphate was used as a pH-responsive mineral on the shell of PNPs. Because of HA affinity with receptors on synovial macrophages, the particles displayed higher accumulation in the inflamed paw of collagen-induced arthritis (CIA) mouse. Jeon et al. reported an HA-5β-cholanic acid conjugate as the potential carrier of MTX to treat RA [123]. Because of the pH-sensitive nature of the conjugate, MTX was rapidly released from PNPs at the inflamed synovial tissue of RA. It is well documented that γ-secretase inhibitors could effectively improve inflammatory arthritis by suppressing the notch signaling pathway. Heo et al. prepared HA-NPs bearing a γ-secretase inhibitor (DAPT) as potential therapeutics for RA [124]. The DAPT loaded HA-NPs exhibited high therapeutic efficacy in a CIA mouse model compared to free DAPT.

## 5. Conclusions

Owing to its appealing physicochemical properties, HA has received increasing attention in biomedical fields. The hydrophilic nature of HA allows prolonged circulation of HA-based NPs in the bloodstream, resulting in their passive accumulation in tissues with leaky blood vessels [60,125]. In addition, HA-based NPs can be used for active targeting of a specific tissue, composed of HA receptor-bearing cells. After reaching the targeted site, burst release of therapeutic or imaging agents and activation of their functions at the disease site may occur in response to Hyals. Inflammatory tissues have hallmarks not found in normal tissue; they often include cells over-expressing HA receptors and have abnormal vasculature with unique microenvironments such as low pH and high level of specific enzymes [90]. Therefore, HA and its derivatives are promising candidates to develop targeted carriers of therapeutic or imaging agents for inflammatory diseases. Moreover, there is growing evidence that, apart from its role as a drug carrier, an empty HA-NP with no drug can exert therapeutic effects in T2D and atherosclerosis by suppressing the proinflammatory response in peripheral tissues by blocking CD44 [67,83]. Thus, self-assembled HA-NP could be a potential therapeutic agent for treatment of not only T2D and atherosclerosis, but also other CD44-mediated inflammatory diseases. In addition, self-assembled HA-NP-bearing drugs might further improve the therapeutic outcome or simultaneous multiple effects, depending on type of drug used.

Even though much effort has been devoted to biomedical applications of HA-NPs, only a few NPs are at the clinical stage. Several limiting issues need to be addressed to minimize the gap between basic research and its clinical translations [126]. The production, synthetic modification, and precise characterization of HA and HA-NPs should be accurately performed in accordance with the clinical requirements. The molecular weight of HA and its CD44 targeting ability are closely connected; similarly, the size of HA-NPs has a marked influence on its pharmacokinetics. Therefore, how HA’s molecular weight in the nanoparticles and the nanoparticle size influences the biological and immunological functions should be thoroughly investigated both in vitro and in vivo. Finally, more research needs to be carried out towards understanding the safety and the clearance of HA-NPs in vivo.
